# Fusion of a Xylan-Binding Module to Gluco-Oligosaccharide Oxidase Increases Activity and Promotes Stable Immobilization

**DOI:** 10.1371/journal.pone.0095170

**Published:** 2014-04-15

**Authors:** Thu V. Vuong, Emma R. Master

**Affiliations:** Department of Chemical Engineering and Applied Chemistry, University of Toronto, Toronto, Ontario, Canada; Iowa State University, United States of America

## Abstract

The xylan-binding module *Clostridium thermocellum* CBM22A was successfully fused to a gluco-oligosaccharide oxidase, GOOX-VN, from *Sarocladium strictum* via a short TP linker, allowing the fused protein to effectively bind different xylans. The presence of the *Ct*CBM22A at the N-terminal of GOOX-VN increased catalytic activity on mono- and oligo-saccharides by 2-3 fold while not affecting binding affinity to these substrates. Notably, both GOOX-VN and its CBM fusion also showed oxidation of xylo-oligosaccharides with degrees of polymerization greater than six. Whereas fusion to *Ct*CBM22A did not alter the thermostability of GOOX-VN or reduce substrate inhibition, *Ct*CBM22A_GOOX-VN could be immobilized to insoluble oat spelt xylan while retaining wild-type activity. QCM-D analysis showed that the fused enzyme remained bound during oxidation. These features could be harnessed to generate hemicellulose-based biosensors that detect and quantify the presence of different oligosaccharides.

## Introduction

Currently, the Carbohydrate Active enZyme database (CAZy, http://www.cazy.org) has categorized carbohydrate-oxidizing enzymes into 11 auxiliary activity (AA) families based on sequence similarities. Several of these AA families are important for plant biomass processing, and some have been used to create biosensors for real-time measurement of cellulase activity on insoluble polymeric substrates [1] or to reduce sugar consumption by lignocellulolytic fungi, maximizing ethanol yields [2]. While many carbohydrate oxidases have been reported to work on mono- or di-saccharides, the members of families AA3_1 [3], AA5_2 [4] and AA7 [5] also show oxidation of larger oligosaccharides and even polysaccharides.

Among those oligosaccharide oxidases are gluco-oligosaccharide oxidases (GOOX, AA7, EC 1.1.3.x), from *Sarocladium strictum* strain CBS 346.70 and strain T1, which have been previously characterized by our group [6], [7] and others [8]-[10], respectively. GOOX contains two distinct structural domains: the F domain recruits the N-terminal residues 1–206 and residues that covalently bind the FAD cofactor while the S domain constitutes most of the carbohydrate-binding groove [11]. The enzyme effectively oxidizes the C1 hydroxyl of a broad range of oligomeric substrates including lactose, malto-, cello- and xylo-oligosaccharides. Structural analyses of GOOX indicate that the oxidization of substituted xylo-oligosaccharides [7] and maltoheptaose [9] is enabled by a comparatively open active site [11]. Among the GOOX variants from *S. strictum* CBS 346.70 is GOOX-VN [6]; the substrate specificity of GOOX-VN is not different from the recombinant wild-type enzyme [7] and the biochemical properties of GOOX-VN have been well characterized [6], [7].

Glucose biosensors account for approximately 85% of the world market for biosensors [12]. With broad substrate specificity, GOOX-based sensors would allow detection of diverse oligosaccharides such as cello-, xylo-, malto-oligosaccharides and lactose generated in biorefinery and food industries. Due to their biocompatibility and film-forming capabilities, polysaccharides such as chitin and chitosan have been widely used for electrochemical biosensor applications [13]. Hemicelluloses represent an immense renewable resource of biopolymers; however, their application potential has been comparatively underexploited [14]. Conductivity of hemicelluloses has not been extensively studied; however, various nanomaterials, including gold nanoparticles and carbon nanotubes has been used as electrical connectors to increase conductivity of other biopolymers in glucose oxidase-based amperometric biosensors [15].

Enzyme immobilization on solid interfaces is one of the key factors for the fabrication of enzyme-based devices [16]. GOOX from the strain T1, GOOX-T1, was previously cross-linked to chitosan using polyethyleneimine and glutaraldehyde for continuous production of acidic oligosaccharides [17]. However, the immobilization efficiency is only 62% [17] and the process requires two steps: 1) enzyme purification and 2) chemical treatment. Alternatively, carbohydrate binding modules (CBMs) can be used to adsorb enzymes to polysaccharides, potentially enabling purification and immobilization in one step. CBMs are currently classified into 68 families in the CAZy database, and are often present as non-catalytic modules of glycoside hydrolases that operate on insoluble, polymeric substrates [18]. Different roles of CBMs have been suggested, for instance, increasing enzyme substrate proximity as well as targeting to specific features of the substrate [18], [19]. Therefore, CBMs have been fused to various hydrolytic enzymes to increase enzymatic activity on soluble [20] and insoluble polymeric substrates [21], [22]. Additionally, due their binding affinity, these non-catalytic modules CBMs have been used for enzyme immobilization [23], [24]. Although all biochemically characterized AA7 members, including GOOX, do not have a native CBM, several putative AA7 enzymes from *Botryotinia fuckeliana* and *Arthrobotrys oligospora* contain CBMs, including CBM1 and CBM18 [25], supporting the feasibility of adding a carbohydrate-binding module to GOOX. Besides promoting enzyme recycling and simplifying downstream processing, the fusion of GOOX and a xylan-binding module is also expected to facilitate the protein purification process.

In the current study, the xylan-binding module *Ct*CBM22A (also known as CBM22-2) from *Clostridium thermocellum* was covalently linked to the N-terminus of GOOX through a TP-rich linker. *Ct*CBM22A was chosen for this analysis since the binding affinity of this CBM has been characterized [26] and its structure has been solved (PDB ID: 1H6Y, 1DYO and 1H6X) [26]. Furthermore, being sourced from a thermophilic organism meant it might confer thermostability to GOOX, as has been observed for CBM addition to other enzymes [20], [22]. The resulting *Ct*CBM22A_GOOOX-VN fusion protein was then characterized in terms of binding affinity, catalytic efficiency, as well as suitability for immobilization. Briefly, the CBM fusion bound well on polymeric substrates, and at least doubled catalytic activity on mono- and oligo-saccharides. The enzyme can be immobilized on hemicellulose and the immobilized enzyme was found suitable for a continuous flow reaction system.

## Materials and Methods

### Materials

*Sarocladium strictum* type strain CBS 346.70 was obtained from the American Type Culture Collection no. 34717. Glucose, xylose, cellobiose, oat spelt xylan, and beechwood xylan were purchased from Sigma (St. Louis, USA), while other cello-oligosaccharides as well as xylo-oligosaccharides were purchased from Megazyme (Wicklow, Ireland). Propoxylated wheat bran hemicellulose and *Thermobifida fusca* xylanase 11A [27] were kindly provided by Prof. Yaman Boluk (University of Alberta, Canada) and Prof. David Wilson (Cornell University, USA), respectively.

### Gene fusion

The *Ct*CBM22A sequence with its native short linker-like (loop) regions (10 amino acids at the N-terminus and 7 amino acids at the C-terminus), together with a TP-rich linker (SRGGGTATPTPTPTPTP) at the C-terminus, was synthesized by NZYTech (Lisboa, Portugal) after codon optimization for *Pichia pastoris* expression. The synthesized CBM sequence was then fused with the GOOX-VN sequence in pPICZαA (Life Technologies, USA). DNA sequences were confirmed at the Center for Applied Genomics (the Hospital for Sick Children, Toronto). The synthetic gene was deposited in the GenBank database under the accession number of KC707633.

### Substrate docking

The GOOX-VN model was built based on the X-ray structure of *S. strictum* strain T1 (PDB ID: 2AXR) using the Swiss-Model Workspace (http://swissmodel.expasy.org) while the non-helical model of the linker was built by MODBASE (http://modbase.compbio.ucsf.edu), based on the X-ray structure of a polysaccharide deacetylase from *Bacillus subtilis* (PDB ID: 1NY1). The docking protocol was previously described [6]. Briefly, the program AutodockTools 1.5.2 ran on Python 2.5 (http://autodock.scripps.edu/) was used to prepare xylohexaose and the enzyme for docking. The program, Autogrid 4, which pre-calculates grip maps of interaction energies, was used to prepare the grid files, and then docking simulation was performed by Autodock 4 (http://autodock.scripps.edu/).

### Recombinant protein expression and purification

The confirmed plasmid was transformed into P. pastoris KM71H following the manufacturer's instructions (Life Technologies, USA). The transformants were screened for protein expression by immuno-colony blot as previously described [6] as well as using an overlay activity assay [7]. Positive transformants, together with the GOOX-VN producing P. pastoris strain, were grown at 15 °C and 250 rpm for 5 days, and 0.5% methanol was added every 24 hr to induce recombinant protein expression. The proteins in culture supernatants were purified following the previously reported method [7]. Briefly, desalted protein samples were mixed with Ni-NTA resin (Qiagen, USA), bound proteins were then eluted with 250 mM imidazole, followed by centrifugal buffer exchange with 50 mM Tris-HCl pH 8.0. Protein concentrations were measured using the Bradford method (Bio-Rad Laboratories, USA), and confirmed by band densitometry on SDS-PAGE gels, where the band density of GOOX variants and the bovine serum albumin (BSA) reference protein were determined using ImageJ (http://rsbweb.nih.gov/ij/).

The proteins were treated with a peptide-N-glycosidase, PNGase F (New England Biolabs, USA) using the same conditions as previously reported [6] for investigating glycosylation.

### Monosaccharide and oligosaccharide oxidation

A 96-well chromogenic assay [7], [8] was used to measure H_2_O_2_, the co-product of carbohydrate oxidation. Briefly, the production of H_2_O_2_ in 50 mM Tris-HCl buffer pH 8.0 was coupled to the oxidation of 4-aminoantipyrine by horseradish peroxidase and measured continuously at 500 nm and 37 °C for 15 min. Michaelis–Menten kinetics and substrate inhibition parameters were determined using the same procedures as previously reported [7]. The temperature stability of enzymes was determined by incubating enzymes for 1 hr at temperatures between 30 and 60°C, and residual activities were then measured with 0.5 mM cellobiose.

### Oxidation of polymeric substrates

The H_2_O_2_ -based colorimetric assay was also used to measure the oxidation of 0.1% polymeric substrates including oat spelt xylan, beechwood xylan and propoxylated wheat bran hemicellulose. The soluble and insoluble fractions of oat spelt xylan were separated by mixing oat spelt xylan (4%, w/v) with 50 mM Tris-HCl pH 8.0 for 48 hr at room temperature and then centrifuging at 4,000 × g for 30 min. Arabinose side chains of soluble oat spelt xylan were removed by incubating 1% of this substrate with 100 nM of *Streptomyces thermoviolaceus* arabinofuranosidase in 50 mM Tris-HCl pH 6.8 at 60°C for 2 hr.

Colorimetric detection of products from soluble substrates were measured every min for 60 min while activity on insoluble substrate was measured at 6 time points over 5 hr. At each time point, the reaction was centrifuged at 13,000 × g for 1 min to precipitate particles. The supernatant was transferred to a 96-well plate, mixed with the chromogenic mixture, and the absorbance at 500 nm was immediately measured. Different concentrations of H_2_O_2_ (up to 150 µM) were incubated with polymeric substrates to generate the standard curve. Activity on polymeric substrates was normalized by molecular weight instead of mass, due to the difference in molecular weight of the two proteins.

### HPAEC-PAD for analyzing oxidized products

Oxidized products from GOOX-VN or *Ct*CBM22_GOOX-VN treatment of xylo-oligosaccharides and polymeric xylan were also characterized by high-performance anion-exchange chromatography with pulsed amperometric detection (HPAEC-PAD) [28]. Reactions containing xylose to xylohexaose, and a mixture thereof, were incubated with 160 nM of *Ct*CBM22_GOOX-VN at 37°C for 16 hr to promote complete oxidation. Reactions containing 0.1% of each xylan sample were similarly incubated with 160 nM of GOOX-VN or CtCBM22A_GOOX-VN, rotated at 8 rpm at 37°C for 16 hr, and then heat-treated at 100°C for 10 min before being treated with 50 nM *T. fusca* endoxylanase Xyn11A [27] in 50 mM Tris-HCl pH 8.0 at 50°C for 16 hr. All samples were then filtered through 0.45 µm centrifugal filter tubes (Corning, USA).

HPAEC-PAD was conducted using an ICS5000 system (Dionex, USA). 10 µL samples in 50 mM NaOH were separated by a CarboPac PA1 2× 250 mm analytical column (Dionex, USA) with the column temperature set at 30 °C. The samples were eluted at 0.25 mL/min with eluent A (0.1 M NaOH) in a linear gradient toward an increasing proportion of eluent B (1 M NaOAc in 0.1 M NaOH). The gradient reached 10% B at 20 min after injection, 20% B at 45 min and 30% B at 50 min. All eluent bottles were connected to a nitrogen gas tank and the pressure was kept at 5 psi. Chromatograms were recorded and analyzed using Chromeleon 7.2 (Dionex, USA).

### Binding affinity on polymeric substrates

Protein binding on soluble oat spelt xylan, beechwood xylan and propoxylated wheat bran hemicellulose was evaluated by affinity gel electrophoresis [29]. Briefly, 0.01% of polysaccharide was added to 7.5% (w/v) acrylamide in 25 mM Tris and 250 mM glycine buffer pH 8.3 prior to polymerization to produce native polyacrylamide gels. 5 µg of GOOX-VN, its CBM fusion as well as BSA were loaded on the gels. Electrophoresis was run using a cold Tris-Glycine buffer pH 8.3.

Binding on insoluble oat spelt xylan was evaluated by comparing the bound and unbound fractions using SDS-PAGE. Specifically, 10 µg of enzyme was mixed with 0.5 mg of insoluble oat spelt xylan for 10 min at 4 °C in 250 µL of a buffer solution (20 mM CalCl_2_, 0.05% Tween 20 and 50 mM Tris-HCl pH 8.0). After centrifugation, the supernatant containing the unbound protein fraction was collected and vacuum-concentrated while the pellets were washed with the buffer solution, and then the bound proteins were extracted with 20 µL of a denaturing solution (10% SDS and 10% β-mercaptoethanol) for 10 min at 100°C. The bound and unbound fractions were then analyzed on 10% SDS-PAGE gels.

### Enzyme immobilization

A 500 µL sample of insoluble oat spelt xylan (1%, w/v) was washed 3 times with the reaction buffer (50 mM Tris-HCl pH 8.0) using 0.45 µm modified nylon centrifugal filter tubes (VWR, USA) and centrifuging at 2,000 × g for 1 min to replace the wash solution. Following three washes to remove mono-sugars and low molecular weigh oligosaccharides, xylan was suspended with the reaction buffer, and then enzyme was added to a final concentration of 80 nM. The enzyme and xylan suspension was mixed in centrifugal filter tubes on a 360° rotator at 8 rpm at room temperature for 10 min, and then washed 3 times by centrifuging at 250 × g for 1 min. The xylan retentate was suspended in 500 µL of the reaction buffer, and then 50 µL of the immobilized enzyme was transferred, using large-orifice pipet tips, to a new 0.45 µm centrifugal filter tube containing 0.5 mM cellobiose, together with the components of the H_2_O_2_ -based colorimetric assay. After incubated at 37°C for 15 min, the reactions were spun down at 250 × g for 1 min, and the absorbance of the flow-through was measured at 500 nm.

To study the stability of the immobilized enzyme in the presence of colorimetric assay reagents, after oxidation and color development, the enzyme with all assay components was kept stored at room temperature on 0.45 µm centrifugal filter tubes. Every 24 hr, the tubes were spun down at 250 × g for 1 min to remove the previous assay components, and then the immobilized enzyme was washed 3 times with 50 mM Tris-HCl pH 8.0 before it was assayed with 0.5 mM cellobiose. This procedure was repeated for 72 hr.

### Quartz crystal microbalance with dissipation (QCM-D)

Hemicellulose films were prepared by spin-coating [30]. The SiO_2_ sensors (Q-Sense, Sweden) were spin-coated with 1% propoxylated wheat bran hemicellulose at 800 rpm for 25 s, 1,200 rpm for 25 s, 1,750 and 2,500 rpm for 30 s, and finally 6,000 rpm for 45 s using a WS-400B-6NPP spin coater (Laurell Technologies, USA). QCM-D experiments were performed using the Q-Sense E4 instrument (Q-Sense, Sweden). The QCM-D principle is described in detail elsewhere [30]. Briefly, when material adsorbs on the surface, the oscillation frequency is decreased; the change from the fundamental frequency (5 MHz, n = 1) and its overtones (n = 3, 5, 7, 11, and 13) is recorded. Damping of the oscillation due to frictional losses by periodically stopping the oscillation of the quartz crystal is presented as dissipation. Viscoelastic materials display faster damping, resulting in higher changes in the dissipation value. The flow rate of the entire experiment was kept constant at 0.1 mL/min and the temperature was maintained at 25 °C. The reaction buffer (50 mM Tris-HCl pH 8.0) was applied to hemicellulose-coated sensors until frequency and dissipation stabilized (approximately 4–5 hr). Once stable, 10 µg/mL of *Ct*CBM22A_GOOX-VN was allowed to flow over the coated sensors until the new frequency and dissipation values stabilized, at which time the protein solution was replaced by the reaction buffer (for washing), and then 0.5 mM cellobiose (for oxidation). The adsorbed layer thickness was calculated by the Q-Tools software (Q-sense, Sweden) using the Voigt model with default parameters.

## Results and Discussion

### Protein fusion construction and production

**Figure 1:**
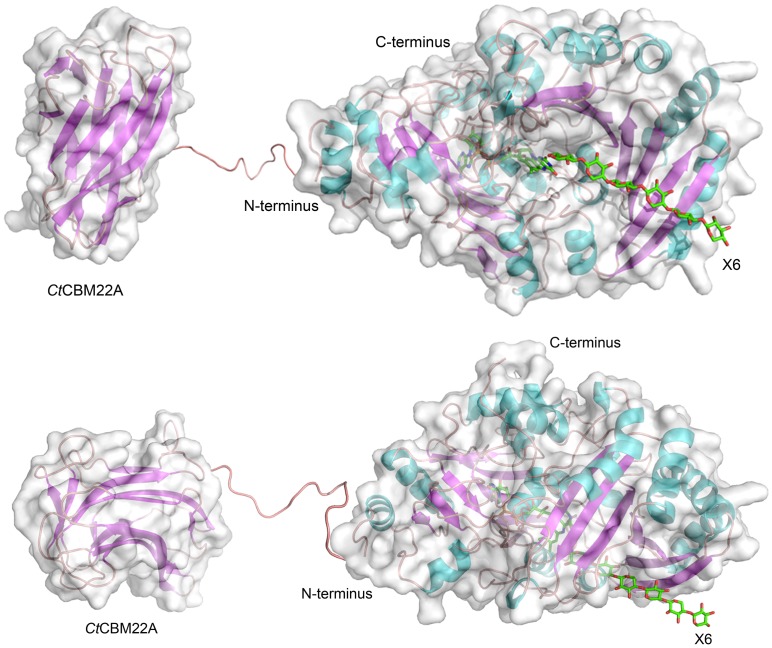
Structural model of the fusion protein. GOOX-VN was fused to *C. thermocellum* CBM22A (PDB ID: 1DYO) via a TP-rich linker. The model of GOOX-VN was built from the X-ray structure of GOOX-T1 (PDB ID: 2AXR) while the non-helical model of the linker was built from the X-ray structure of a *Bacillus subtilis* polysaccharide deacetylase (PDB ID: 1NY1). Xylohexaose (X6) was docked into the active site of GOOX-VN using Autodock 4. Top: the substrate-entrance view of the fused protein model; Bottom: the side view of the fused protein model.

Both termini of GOOX-VN orient in the same direction (Fig. 1); however, while the C-terminus is positioned behind the active site, the N-terminus is positioned on the same planar surface as the substrate binding site. Accordingly, designs for the fusion protein linked the CBM22A and the TP-rich linker to the N-terminus of GOOX-VN (Fig. 1). In an effort to increase the yield of recombinant enzyme from *P. pastoris*, the induction temperature was reduced from 30°C to 15°C, which boosted the yield of purified GOOX-VN more than 20 times, generating 200 mg of the purified protein/L. Similar yields were obtained for *Ct*CBM22_GOOX-VN. Proteins were purified with over 98% purity, as estimated by SDS-PAGE (Fig. S1).

SDS-PAGE analysis of the fused protein suggested *Ct*CBM22A_GOOX-VN was heavily glycosylated (Fig. S1). The electrophoretic mass of the purified fusion protein was above 100 kDa (Figs. S1 and 5) while its predicted mass is 76 kDa. GOOX-VN itself has been shown to be N-glycosylated, increasing the molecular weight by approximately 20% [6], [7]. Therefore, besides the N-glycosylation in GOOX-VN, this increased mass is likely due to N- and/or O-glycosylation in the CBM and linker. PNGase F treatment confirmed N-glycosylation in the CBM fusion (Fig. S1). There is one potential N-glycosylation site (N50) and one predicted O-glycosylation site (T160) in the *Ct*CBM22A module, as well as six predicted O-glycosylation positions in the linker sequence (as predicted by NetNGlyc 1.0 Server and NetOGlyc 3.1 Server, www.cbs.dtu.dk/services/). *P. pastoris* has been reported to secret proteins with mannose-containing O-glycans [31], [32]. Notably, glycosylation at N50 and T160 in the CBM is unlikely to affect binding, as these positions are not close to the binding surface of the *Ct*CBM22A.

### Increase in oligosaccharide oxidation

*Ct*CBM22A_GOOX-VN showed similar *k*_cat_ for monosaccharides (glucose and xylose) and their oligosaccharides with a degree of polymerization (DP) of 2 to 6; however, corresponding *K*_m_ values for these two groups of substrate were up to 3 orders of magnitude different: *K*_m_ on xylose was 135 mM while that for xylo-oligosaccharides was approximately 0.1 mM (Table 1). Therefore, it is likely that the low catalytic efficiency of *Ct*CBM22A_GOOX-VN on glucose and xylose is mainly due to weak binding affinity to those monosaccharides. *K*_m_ values between oligosaccharides were not significantly different, further supporting the existence of two binding subsites near the active site of GOOX-VN [11]. Compared with the kinetics data of GOOX-VN alone [7], the presence of the CBM and linker significantly increased *k*_cat_ on glucose, xylose and their oligosaccharides by more than 2-fold (Table 1). For instance, the *k*_cat_ of GOOX-VN and *Ct*CBM22A_GOOX-VN on xylobiose is 449 [7] and 1,350 min^−1^, respectively.

**Table 1:** Kinetic parameters and substrate inhibition of *Ct*CBM22A_GOOOX-VN on cello-oligosaccharides and xylo-oligosaccharides.

	Kinetic parameters*			Substrate inhibition		
	*k*_cat_ (min^−1^)	*K*_m_ (mM)	*k*_cat_/*K*_m_ (mM^−1^ min^−1^)	*V*_i_/*V*_max_ (%)	*K*_i_ (mM)	*n* ^H^ **
Glucose	1,090 ± 16	11 ± 1	95 ± 7	–	–	–
Cellobiose	1,148 ± 5	0.060 ± 0.001	19,300 ± 300	45	2.9 ± 0.3	1.3
Cellotriose	1,210 ± 60	0.09 ± 0.01	12,800 ± 1,900	50	1.88 ± 0.16	1.5
Cellopentaose	1,080 ± 30	0.08 ± 0.01	14,100 ± 1,800	56	2.2 ± 0.3	1.5
Cellohexaose	1,190 ± 40	0.13 ± 0.01	9,000 ± 1,000	62	1.4 ± 0.3	1.3
Xylose	1,230 ± 20	135 ± 8	9 ± 1	–	–	–
Xylobiose	1,350 ± 30	0.09 ± 0.01	14,500 ± 1,400	75	8 ± 2	1.5
Xylotriose	1,330 ± 30	0.12 ± 0.01	11,000 ± 1,000	71	6 ± 3	1.2
Xylopentaose	1,630 ± 50	0.10± 0.01	15,700 ± 1,900	–	–	–
Xylohexaose	1,340 ± 40	0.12 ± 0.02	11,500 ± 1,600	–	–	–

^*^Data are mean values ± standard errors; 16 nM of enzyme was used in each reaction.

^**^Hill coefficient, values greater that one indicate positive cooperative binding.

– No inhibition detected with used substrate concentrations.

*Ct*CBM22A binds tightly to xylo-oligosaccharides, from xylobiose to xylohexaose, with *K*_a_ of 1.5×10^5^ M^−1^ for xylohexaose [26], and exhibits maximum affinity for xylo-oligosaccharides containing four or more xylose units [26]. However, *K*_m_ values of the fused protein and the wild type on xylo-oligosaccharides were not significantly different: *K*_m_ of GOOX-VN on xylohexaose is 0.10 ± 0.01 mM [7] while that of *Ct*CBM22A_GOOX-VN is 0.12 ± 0.02 mM (Table 1). These results indicate that sugar binding by the CBM did not compete with substrate binding in the active site groove of GOOX-VN, and that the increased activity of the fused protein on oligosaccharides was unlikely directly attributed to substrate binding by the CBM.

Alternatively, the addition of the linker and the CBM at the N-terminus might impact metal binding by residues in the N-terminus including Glu16, Glu17 and Asp36 [7]. In particular, it is conceivable that the relatively rigid TP-rich linker caused local conformational change in the FAD-binding domain, as proposed for the V38A mutation at the N-terminal region of GOOX-VN, which also increases *k*_cat_ on glucose, xylose and their oligosaccharides by up to 2-fold [7].

### No effect on substrate inhibition and thermostability

**Figure 2:**
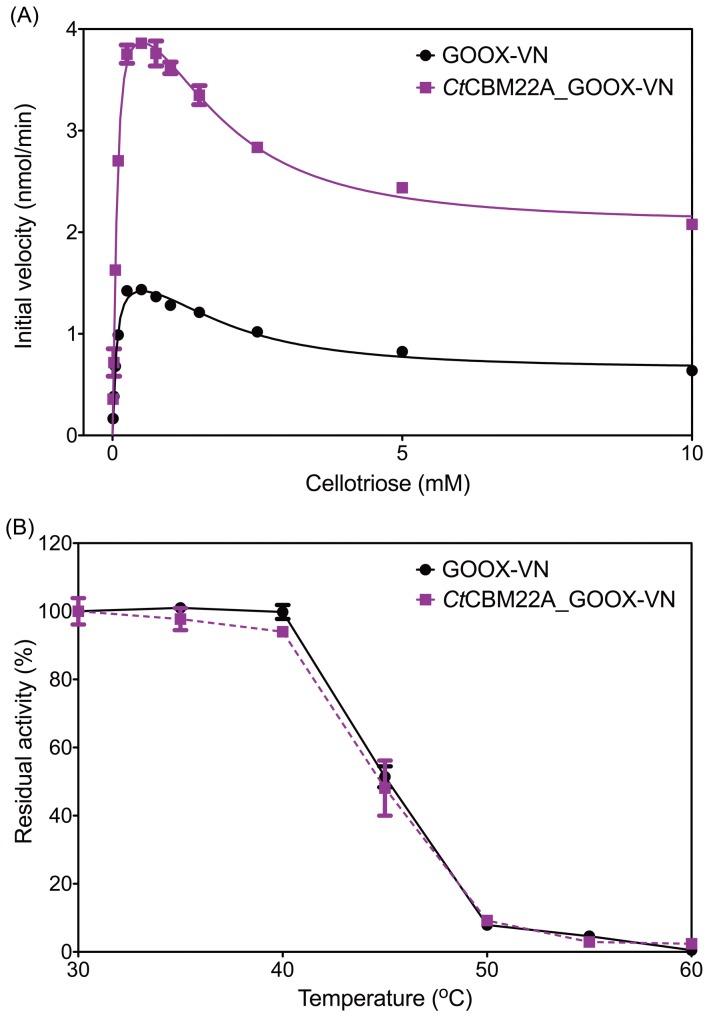
Substrate inhibition and thermostability of GOOX-VN and *Ct*CBM22A_GOOX-VN. (A): The activity of 16 nM of each enzyme on different concentrations of cellotriose, up to 10 mM; (B): Enzymes were incubated for 1 hr at temperatures from 30 to 60°C, and then the residual activities of 16 nM enzyme on 0.5 mM cellobiose were measured.

Substrate inhibition was previously observed with GOOX-VN lacking a CBM [7]. Similarly, oligosaccharide inhibition was observed for *Ct*CBM22A_GOOX-VN (Table 1; Fig. 2A). This result was not surprising since the CBM was fused directly to the FAD-binding domain and not the substrate-binding domain. The inhibitory effect of cello-oligosaccharides (*K*_i_ from 1.4 to 2.9 mM) on the activity of the fusion enzyme was also higher than that of xylo-oligosaccharides (*K*_i_ from 6 to 8 mM) (Table 1), suggesting that these oligosaccharides have different binding affinities for the enzyme-substrate complex. Despite originating from a thermophilic bacterium, and the precedence of CBM addition to increase the thermostability of fused enzymes in some cases [20], [22], the addition of *Ct*CBM22A did not increase the thermostability of GOOX-VN, and both GOOX-VN and the corresponding fusion protein retained half of their activity after 1 hr incubation at 45°C (Fig. 2B).

### Oxidation of larger oligosaccharides and polymeric substrates

**Figure 3:**
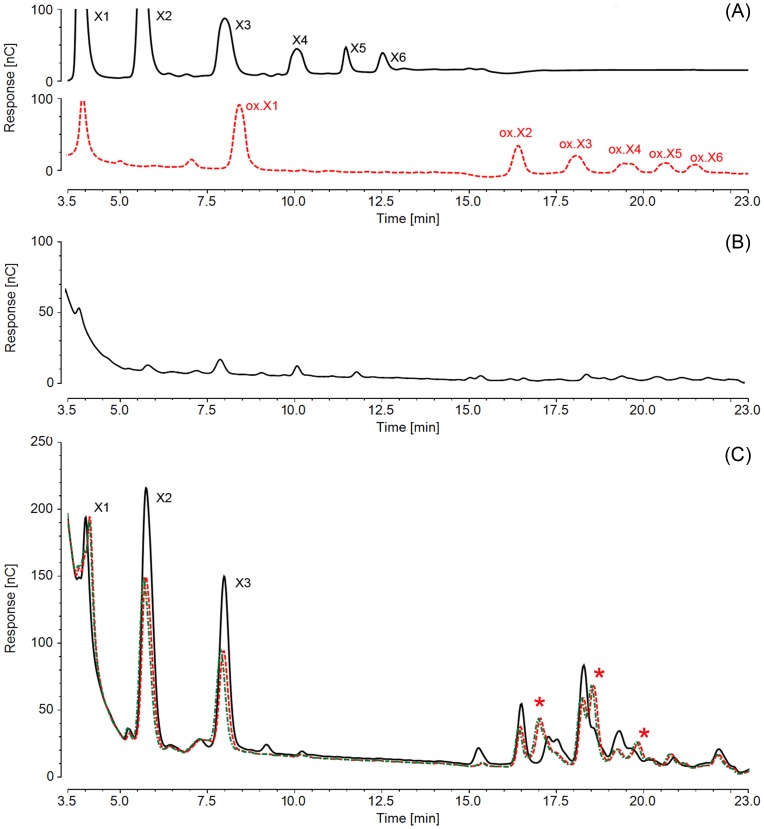
HPAEC-PAD separation of native and oxidized xylo-oligosaccharides. (A): Xylo-oligosaccharide standard from xylose to xylohexaose (X1 to X6, respectively) before oxidation (top) and after the oxidation of *Ct*CBM22A_GOOX-VN, which released oxidized xylose to xylohexaose (ox.X1 to ox.X6, respectively) (bottom). (B): A chromatogram of soluble oat spelt xylan without any enzyme treatment. (C): Xylanase treatment of soluble oat spelt xylan (black solid), GOOX-VN treated soluble oat spelt xylan (red dashed) and *Ct*CBM22A_GOOX-VN treated soluble oat spelt xylan (green dotted dashed); peaks formed by oxidation were asterisked.

HPAEC-PAD analysis of xylo-oligosaccharide oxidation by *Ct*CBM22A_GOOX-VN showed that oligosaccharides with DP of 2–6 were completely oxidized and eluted after 15 min, allowing clear separation from the neutral sugars (Fig. 3A). Therefore, this HPLC approach was used to characterize enzymatic oxidation of xylan and confirm that oxidation occurred on the polymeric fraction of the xylan preparation, rather than low molecular weight oligosaccharides. Briefly, after oxidation by GOOX-VN or *Ct*CBM22_GOOX-VN, xylan samples were heat-treated to denature the oxidases, and then treated with a xylanase before being analyzed by HPLC. *Thermobifida fusca* xylanase Xyn11A was chosen for this analysis, since this endoxylanase efficiently hydrolyzes oat spelt xylan at pH 5 to 9 [27], which is compatible with the buffer conditions used for the oxidation step (pH 8.0). Xylose, xylobiose and xylotriose were clearly observed from xylanase treatment of non-oxidized oat spelt xylan (Fig. 3C, solid line), whereas xylanase treatment of oxidized xylan generated less xylobiose and xylotriose and instead led to three new peaks not found in the non-oxidized sample or among the oxidized xylo-oligosaccharide standards (Fig. 3C). These results suggest oxidation of xylo-oligosacccharides apart from the neutral linear ones up to a DP of 6. To our knowledge, this is the first report to confirm the activity of GOOX-VN as well as *Ct*CBM22_GOOX-VN on soluble oat spelt xylan (Table 2).

**Table 2:** Activity of GOOX-VN and *Ct*CBM22A_GOOOX-VN on hemicelluloses.

	Oxidase activity (µmol/min/µmol)*	
	GOOX-VN	*Ct*CBM22A_GOOOX-VN
Soluble oat spelt xylan	37 ± 1	47 ± 1
Debranched soluble oat spelt xylan	44 ± 4	56.7 ± 0.4
Insoluble oat spelt xylan	–	–
Debranched insoluble oat spelt xylan	–	–
Beechwood xylan	–	–
Propoxylated wheat bran hemicellulose	–	–

^*^Equal molar equivalents were used. 32 nM of each enzyme was assayed with 0.1% substrate.

– No activity was detected.

Activity of the fused protein on polymeric substrates was not increased or detected in the presence of BSA (0.05, 0.1, 0.4 and 0.8%) or CaCl_2_ (10, 20, 40 and 80 mM), which were added to prevent non-specific binding or to tentatively support CBM stability [26], respectively. At present, only two carbohydrate oxidases, galactose 6-oxidase (AA5_2, EC 1.1.3.9) [4] and *Microdochium nivale* carbohydrate 1-oxidase (AA7, EC 1.1.3.x) [5] were reported to show activity on polymeric substrates. Notably, the best polymeric substrate of *M. nivale* carbohydrate 1-oxidase is carboxymethyl cellulose, where its activity on 1% carboxymethyl cellulose is 12.9 min^−1^, which is 9% of its activity on cellobiose [5] and lower than activity of GOOX-VN on soluble oat spelt xylan (Table 2).

A challenge when working with hemicellulose is the presence of different side groups; GOOX variants produced to date show lower activity on branched and substituted xylo-oligosaccharides [7]. Soluble oat spelt xylan contains about 12% arabinose [33], but arabinose is not a sugar substrate of GOOX-VN [6]. Soluble oat spelt xylan was, therefore, treated with a *S. thermoviolaceus* arabinofuranosidase, which is able to remove singly substituted arabinose from oat spelt xylan (unpublished data). The oxidation of both GOOX-VN and its CBM fusion on debranched soluble oat spelt xylan was slightly increased, up to 20% (Table 2), even though doubly substituted arabinose would remain (unpublished data). Overall, commercial beechwood glucuronoxylan is less branched than oat spelt xylan [34], [35]; however, no activity on beechwood xylan was detected. This result could reflect the presence of the xylan reducing sequence (D-Xyl*p*-β(1→4)-D-Xyl*p*-β(1→3)-L-Rha*p*-α(1→2)-D-Gal*p*A-α(1→4)-D-Xyl*p*) identified in dicot plants, which is not known to exist in monocots (reviewed in [36]). Alternatively, the occurence of charged substitutions near the reducing end of glucuronoxylan could reduce GOOX-VN activity, given the comparatively high *K_m_* of the enzyme on a 4-*O-*methyl-α-D-glucuronic acid substituted xylo-oligosaccharide [7].

### Increased hemicellulose binding

**Figure 4:**
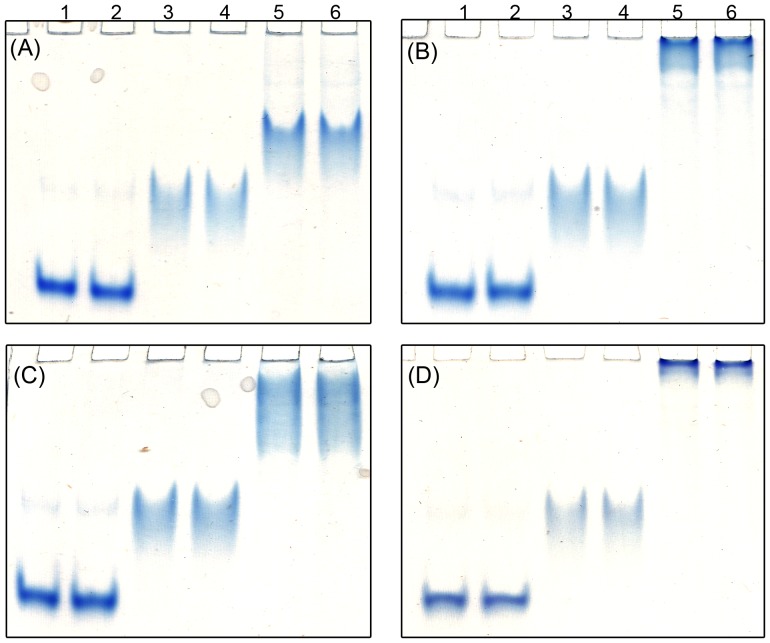
Binding of GOOX-VN and its CBM fusion on soluble polymeric substrates. (A): Control, no polymeric substrate; (B): 0.01% soluble oat spelt xylan; (C): 0.01% beechwood xylan; (D): 0.01% propoxylated wheat bran hemicellulose. Lanes 1 and 2: BSA as the negative control; lanes 3 and 4: GOOX-VN; lanes 5 and 6: *Ct*CBM22A_GOOX-VN.

**Figure 5:**
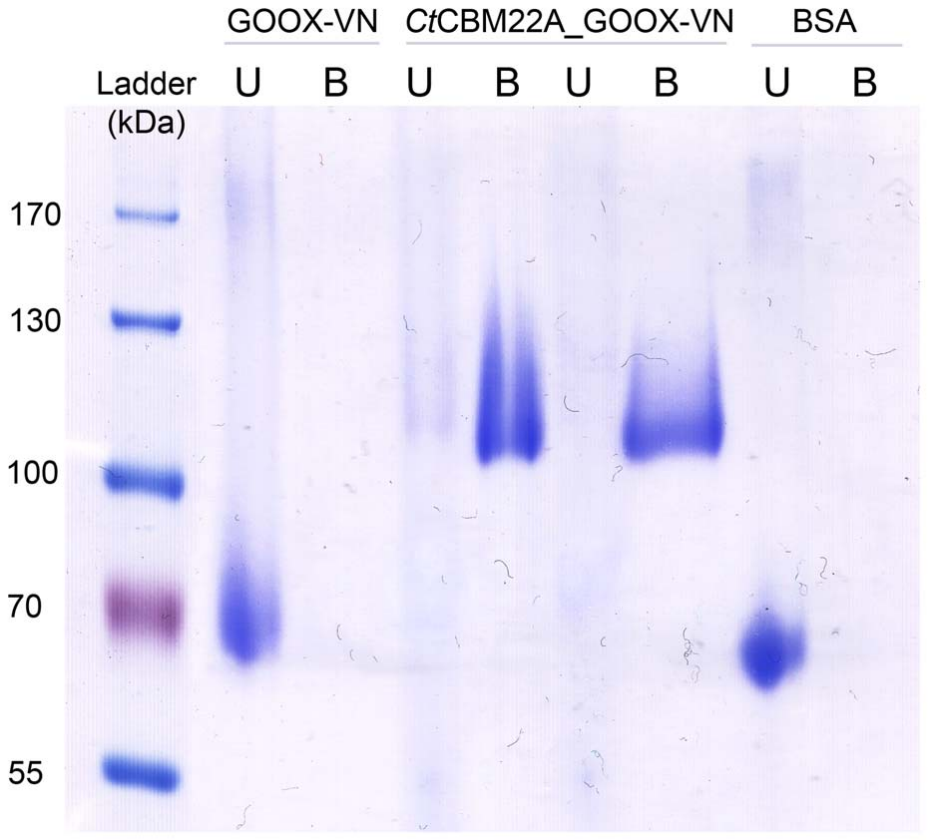
Binding of GOOX-VN and its CBM fusion on insoluble oat spelt xylan. 10 µg of protein was mixed with 0.5 mg of insoluble oat spelt xylan, and then the bound and unbound protein fractions were separated and analyzed using 10% SDS-PAGE gels. U: unbound faction; B: bound faction; BSA was used as the negative control.

The migration of *Ct*CBM22_GOOX-VN through acrylamide gels was significantly retarded by the inclusion of soluble hemicellulose while the electrophoretic mobility of BSA and GOOX-VN was not affected by the presence of these polysaccharides (Fig. 4). In particular, in gels embedded with fully soluble oat spelt xylan and propoxylated wheat bran hemicellulose, the fused protein was nearly immobilized. Furthermore, when incubated with insoluble oat spelt xylan, *Ct*CBM22_GOOX-VN was found only in the bound fraction, whereas GOOX-VN alone was only detected in the unbound fraction (Fig. 5). These findings are consistent with the high binding affinity of *Ct*CBM22A on xylan: its *K*_a_ values on oat spelt xylan and wheat arabinoxylan are 7.6×10^4^ and 8×10^4^ M^-1^, respectively [26]. This analysis also demonstrates that fusion to *Ct*CBM22 promoted enzyme binding to polymeric substrates and that any potential post-translational modification on the *Ct*CBM22A during expression in *P. pastoris* did not eliminate its binding properties. Notably, activity studies using polymeric forms of xylan showed that substrate binding by *Ct*CBM22A did not enhance nor reduce GOOX-VN activity on polymeric substrates. This result suggests that substrate binding alone was not rate limiting and that further modifications of the fusion enzyme should aim to fine-tune xylan positioning within the GOOX-VN active site.

### Immobilization on hemicellulose-based materials

**Figure 6:**
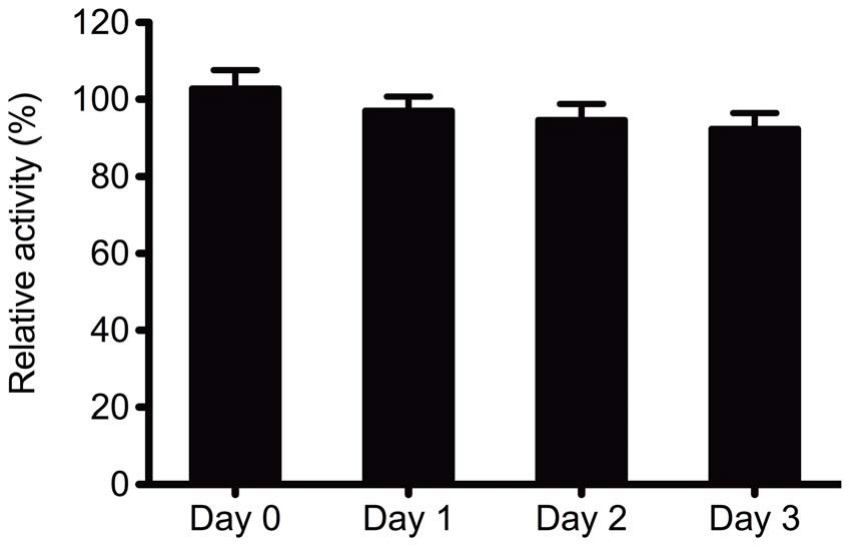
Stability of the immobilized CBM fusion at room temperature. *Ct*CBM22A_GOOX-VN was kept at room temperature with all reaction components, and then its activity on 0.5 mM cellobiose was measured every 24 hr. Relative activity (%) of the immobilized enzyme to the free form in the same assay conditions was shown.

Strong binding of *Ct*CBM22_GOOX-VN to xylans makes this enzyme fusion a good candidate for immobilization on hemicellulose-based chromatographic beads or films for purification and biosensor applications, respectively. In addition to enzyme immobilization, other criteria relevant to these applications are 1) activity after immobilization, and 2) enzyme stability after immobilization and incubation with reaction products. To evaluate whether bound *Ct*CBM22_GOOX-VN was functional, the enzyme fusion was immobilized to insoluble oat spelt xylan, and then activity was measured using cellobiose. Comparisons with equal molar quantities of fresh, free enzyme confirmed that immobilization did not affect the activity of *Ct*CBM22_GOOX-VN (Fig. 6), suggesting that enzyme purification based on xylan binding, along with preparation of materials containing immobilized enzyme, could be carried out in a single step.

The stability of immobilized *Ct*CBM22_GOOX-VN at room temperature and in the presence of reaction products was then assessed by replacing oxidized cellobiose and H_2_O_2_ every 24 hr with fresh cellobiose, and measuring residual enzyme activity. More than 90% of *Ct*CBM22_GOOX-VN activity was retained after three replacements of reaction products with fresh reactants over 72 hr (Fig. 6), suggesting that it is feasible to reuse the immobilized enzyme, and that it would be stable in batch reaction systems.

**Figure 7:**
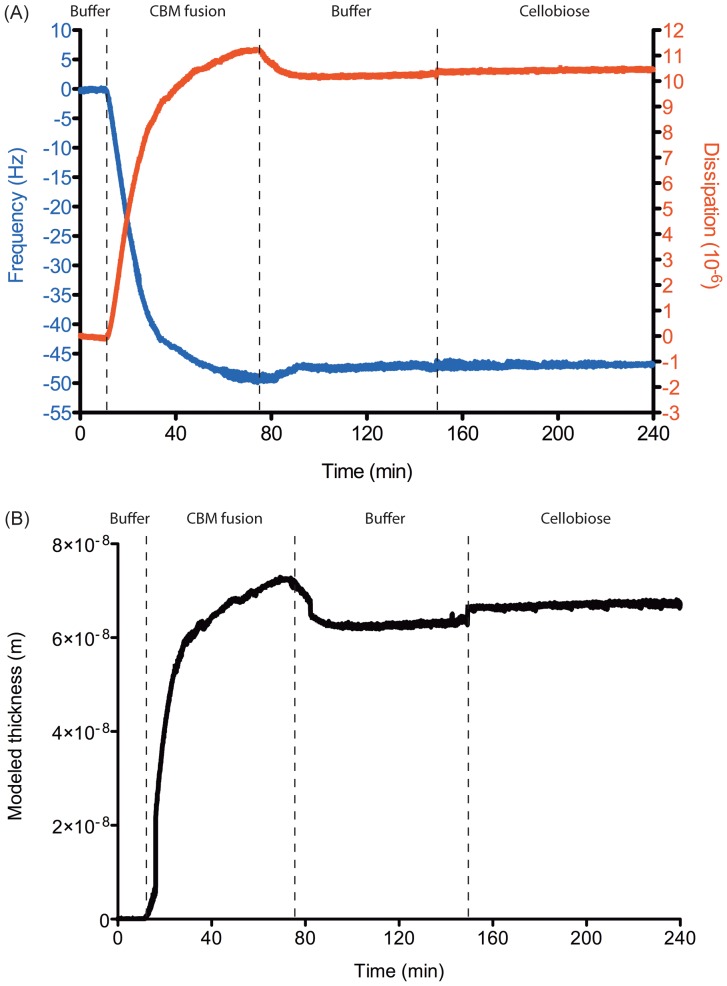
QCM-D responses on binding and activity of the CBM fusion. (A): After the frequency and dissipation responses (n = 5) of xylan-coated sensors were stable in the reaction buffer (50 mM Tris-HCl pH 8.0), the sensors were exposed to 10 µg/mL *Ct*CBM22A_GOOX-VN, followed again with the reaction buffer, and then 0.5 mM cellobiose. (B): The thickness of the adsorbed layers was calculated using the Voigt model provided in the Q-Tools program (Q-sense, Sweden). The thickness values should be considered on a relative basis and should not be considered absolute values as the fitting includes approximations of layer density properties [30].

QCM-D analysis was used to assess the suitability of immobilized *Ct*CBM22_GOOX-VN in continuous-flow systems. QCM-D sensors were coated with propoxylated wheat bran xylan given the comparatively high water solubility of this hemicellulose sample and earlier results confirming *Ct*CBM22_GOOX-VN binding to propoxylated wheat bran xylan (Fig. 4). Binding of *Ct*CBM22_GOOX-VN to the xylan-coated sensors was immediate, increasing both the thickness and viscoelasticity of the surface layer (Fig. 7) Washing with sample buffer resulted in only minor reductions to the surface layer, indicating that the majority of bound *Ct*CBM22_GOOX-VN remained attached to the xylan coated sensor (Fig. 7). Moreover, the enzyme remained bound while catalyzing the oxidation of 0.5 mM cellobiose; where cellobiose oxidation was confirmed by detecting H_2_O_2_ in the flow-through from the QCM-D using the standard colorimetric assay (data not shown).

## Conclusion

The addition of *Ct*CBM22A to GOOX-VN did not alter thermostability, substrate inhibition, or GOOX-VN binding affinity towards glucose, xylose and their oligosaccharides up to a DP of 6; however, *Ct*CBM22A fusion increased the catalytic activity of GOOX-VN on these substrates by more than 2 fold. The CBM fusion along with the wild-type enzyme was shown to oxidize xylo-oligosaccharides from soluble oat spelt xylan with a DP greater than 6. The presence of *Ct*CBM22A allowed the fusion protein to strongly bind both soluble and insoluble xylan, allowing the *Ct*CBM22A_GOOX-VN to be immobilized on insoluble oat spelt xylan without significant loss of enzyme quantity or activity. The immobilized enzyme can be suitable for use in both batch and continuous-flow systems for detecting oligosaccharides and producing acidic forms thereof.

## Supporting Information

Figure S1 **12% SDS-PAGE gel of purified and N-deglycosylated proteins.** Lane 1: 2.5 µL of the PNGase F stock (NEB, 500 U/µL); lanes 2 and 3: purified GOOX-VN (deduced molecular weight of 56 kDa) and its PNGase F-treated form, respectively; lanes 4 and 5: *Ct*CBM22A_GOOX-VN (deduced molecular weight of 76 kDa) and its PNGase F-treated form, respectively.
(TIF)
