# Supplementary figures and images for: Fusion of a Xylan-Binding Module to Gluco-Oligosaccharide Oxidase Increases Activity and Promotes Stable Immobilization
Source: PLoS One. 2014 Apr 15;9(4):e95170. doi: 10.1371/journal.pone.0095170 (PMC3988151; doi:10.1371/journal.pone.0095170)

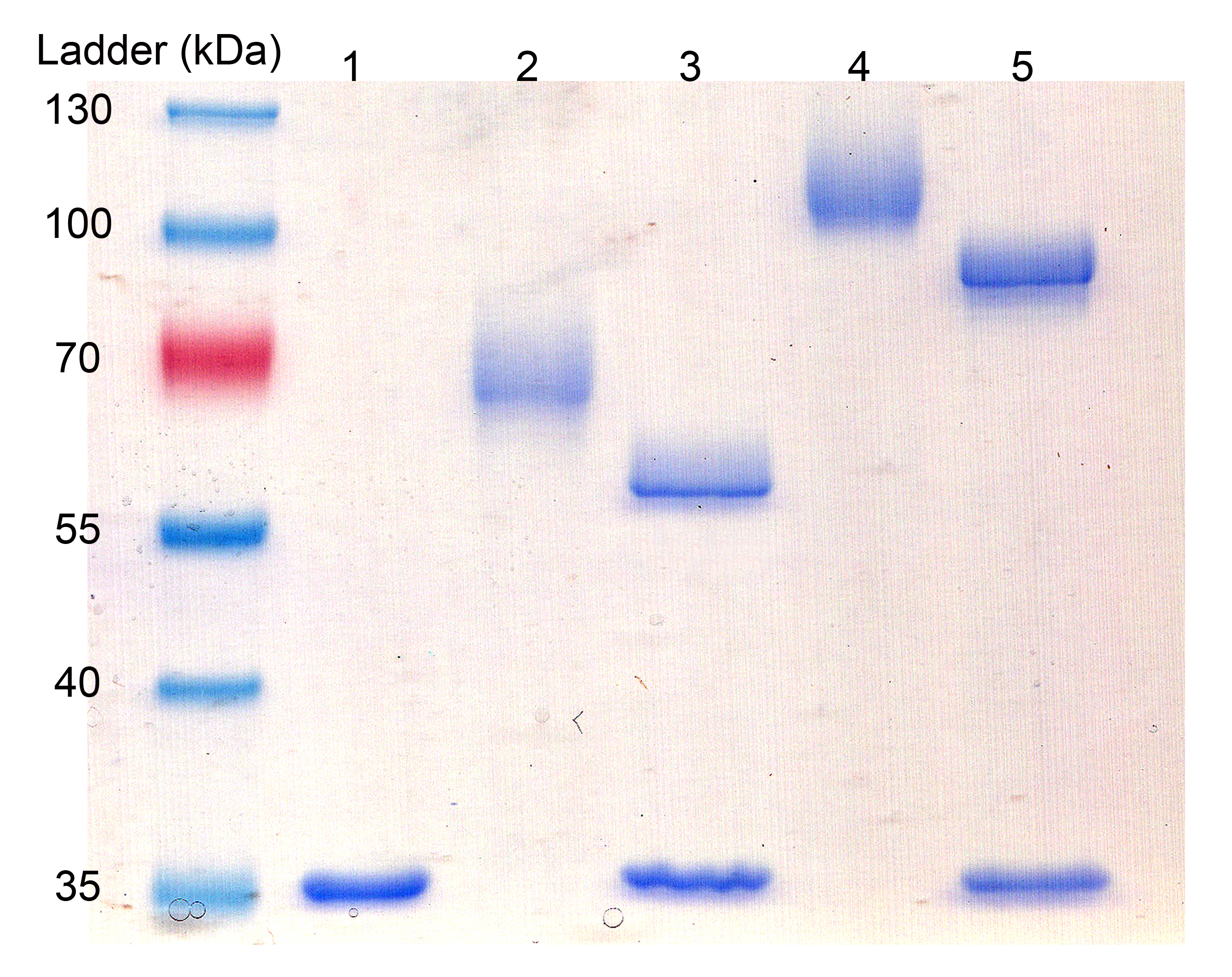

Supplement: Figure S1 — 12% SDS-PAGE gel of purified and N-deglycosylated proteins. Lane 1: 2.5 µL of the PNGase F stock (NEB, 500 U/µL); lanes 2 and 3: purified GOOX-VN (deduced molecular weight of 56 kDa) and its PNGase F-treated form, respectively; lanes 4 and 5: CtCBM22A_GOOX-VN (deduced molecular weight of 76 kDa) and its PNGase F-treated form, respectively. (TIF) [file pone.0095170.s001.tif]
